# Circulating Tumor DNA as a Marker of Recurrence Risk in Stage III Colorectal Cancer: The α-CORRECT Study

**DOI:** 10.1002/jso.27989

**Published:** 2025-01-25

**Authors:** Brenda Diergaarde, Greg Young, David W. Hall, Amin Mazloom, Gina L. Costa, Soma Subramaniam, Melanie R. Palomares, Jorge Garces, Frederick L. Baehner, Robert E. Schoen, Exact Sciences MRD Group

**Affiliations:** 1 Department of Human Genetics, School of Public Health, University of Pittsburgh, UPMC Hillman Cancer Center, Pittsburgh, Pennsylvania, USA; 2 Exact Sciences Corporation, Madison, Wisconsin, USA; 3 Department of Medicine, Division of Gastroenterology, Hepatology and Nutrition, and Department of Epidemiology, University of Pittsburgh, Pittsburgh, Pennsylvania, USA

**Keywords:** colorectal cancer, ctDNA, molecular residual disease (MRD), tumor-informed

## Abstract

**Background and Objectives:**

Identification of colorectal cancer (CRC) patients at high risk of recurrence could be of substantial clinical use. We evaluated the association of ctDNA status, using a tumor-informed assay, with recurrence-free survival (RFS).

**Methods:**

Stage III CRC patients were enrolled between 2016 and 2020. Tumor tissue and serial (every 3 months for years 1–3, biannually for years 4–5) blood samples were collected. Utilizing whole-exome sequencing and selection of 50–200 variants for tumor informed assays, ctDNA status was determined using plasma cell-free DNA.

**Results:**

Of 137 patients enrolled, 124 with 1029 ctDNA results were included in the analyses. Median follow-up was 4.8 years. Plasma ctDNA status was strongly associated with risk of recurrence during the surveillance period (hazard ratio (HR) 49.6, 95% CI: 16.6–148.3; *p* < 0.0001), and at the postsurgical (HR 9.6, 95% CI: 3.2–29.5) and postdefinitive therapy timepoints (HR: 16.7, 95% CI: 6.9–40.3). The estimated 3-year RFS for ctDNA positive and ctDNA negative patients were, respectively, 54.5% and 96.1% after surgery, and 18.2% and 90.0% after definitive therapy. Multivariable analysis indicated ctDNA but not CEA was strongly prognostic for recurrence.

**Conclusions:**

Our tumor-informed ctDNA assay was strongly prognostic for recurrence in patients with stage III colorectal cancer at all timepoints.

## 1 | Introduction

Colorectal cancer (CRC) is a major cause of cancer-related morbidity and mortality worldwide [1]. The current standard of care for stage III colon cancer is surgery followed by adjuvant chemotherapy with an oxaliplatin-based regimen. Surgery alone cures 50%–70% of patients with stage III colon cancers [2]. Adjuvant chemotherapy with oxaliplatin-based chemotherapy cures an additional 6%–12% of patients [3, 4]. However, benefits of adjuvant chemotherapy vary between patients, and therapy-associated toxicity is a significant concern [5, 6]. Accurately identifying patients who have a higher risk of recurrence and are more likely to benefit from adjuvant chemotherapy would be of substantial clinical utility. Early identification of impending colon cancer recurrence may allow for additional or alternative curative interventions.

There has been tremendous interest in the development of blood-based biomarkers in postoperative CRC patients to prognosticate risk of recurrence. Molecular residual disease (MRD) assays have shown that detection of circulating tumor DNA (ctDNA) is independently associated with a high risk of recurrence [7–14]. Several studies have demonstrated that ctDNA positivity is a superior prognostic biomarker to carcinoembryonic antigen (CEA) level and conventional clinicopathological features. Furthermore, monitoring for ctDNA presence during surveillance of patients with resected colon cancers has a reported lead-time benefit of 5.5–8.7 months over radiological recurrence [7–10, 12–14]. However, sensitivities and specificities vary widely across assays and across studies [15], suggesting the need to develop assays with more robust performance.

In the α-COloRectal cancer study to predict REcurrence using Circulating Tumor DNA (CORRECT) study, we evaluated the performance of a patient-specific, tumor-informed MRD assay as a prognostic biomarker for recurrence in patients with stage III CRC. The primary aim was to validate the association between ctDNA detection from each patient’s original tumor in plasma during the surveillance period post-definitive therapy (defined as after adjuvant treatment, or after surgery if no adjuvant treatment was given) and the risk of recurrence.

## 2 | Methods and Materials

### 2.1 | Study Design and Participants

The study cohort consisted of 137 patients with stage III CRC who enrolled into an observational prospective study focused on the evaluation of ctDNA as a prognostic marker for disease recurrence (ClinicalTrials.gov #: NCT02842203). Between 2016 and 2020, a total of 137 recently diagnosed stage IIIA-C CRC patients were enrolled across 19 hospitals in western Pennsylvania. All subjects provided written informed consent, and the study was approved by the University of Pittsburgh Institutional Review Board (STUDY19070371) and, for Exact Sciences, by Advarra (Protocol 00064238). Key eligibility criteria included a recent diagnosis of stage IIIA-C CRC, age ≥ 18 years, and ability to understand informed consent. To produce a patient-specific, tumor-informed MRD assay, a tumor tissue sample, from biopsy or surgery, is required for each patient. One of the 137 patients had no tumor block available, and two nonsurgical patients with rectal cancer had insufficient tumor for sequencing, leaving 134 patients for the current study. The study followed the Strengthening the Reporting of Observational Studies in Epidemiology reporting guidelines [16].

### 2.2 | Sample Collection

**FIGURE 1 |:**
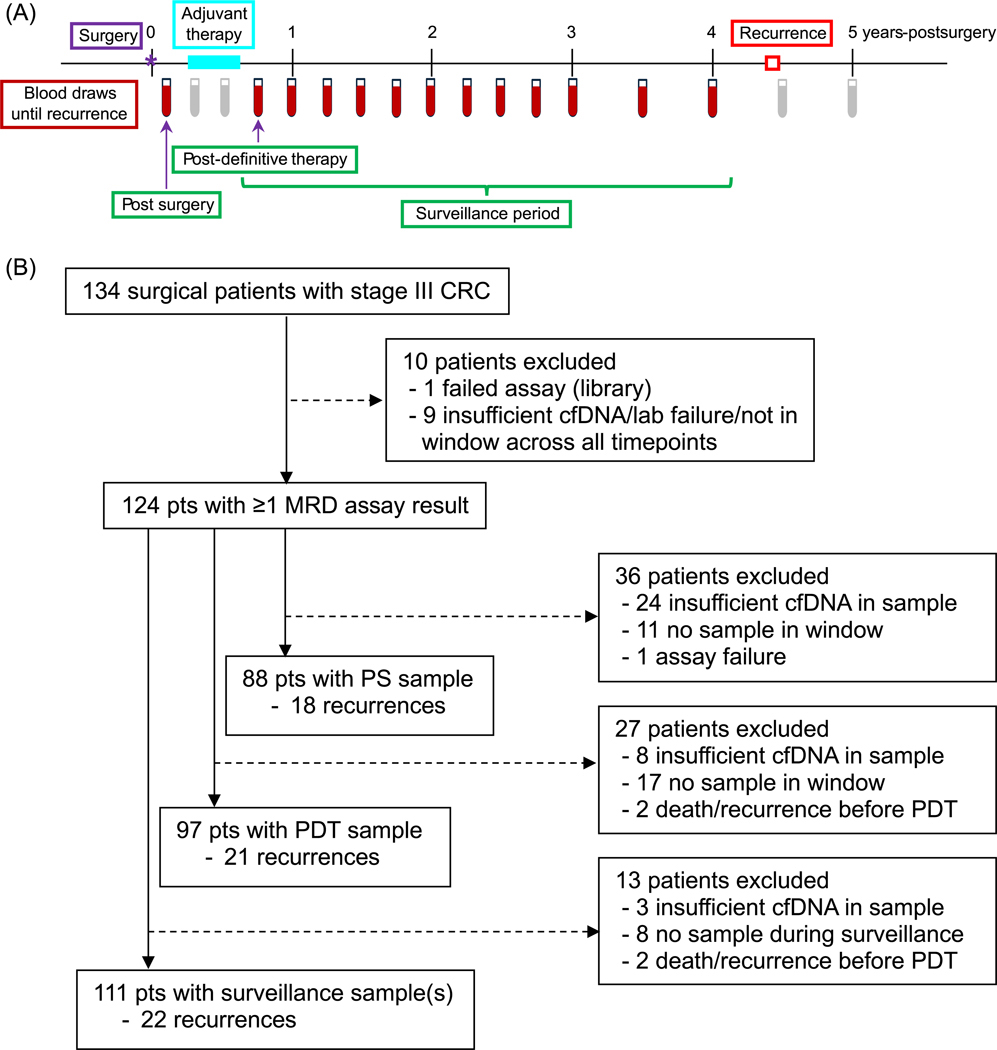
Study overview showing sampling timepoints (A) and number of patients with each outcome (B). (A) Blood draws utilized for the current study are in red. Green boxes represent the three timepoints used for ctDNA analyses. The surveillance period timepoint included ≥ 1 sample, while postsurgical (PS) and post-definitive therapy (PDT) timepoints included a single sample. Samples collected during adjuvant therapy or after recurrence were not utilized and are shown in gray. (B) CONSORT diagram.

Up to 50 mL of blood was collected serially in Streck Cell-Free DNA BCT tubes after surgery, quarterly for the first 3 years after enrollment, and semiannually during years four and five. Three sample timepoints were used for analyses: the single sample postsurgical (PS) and postdefinitive therapy (PDT) timepoints, and the surveillance period, which included the PDT and all subsequent samples (Figure 1A). To be included, the PS sample had to be collected 21–84 days (3–12 weeks) after surgery and before initiation of adjuvant therapy. The PDT sample was defined as the first blood sample collected after the end of adjuvant therapy, or at least 21 days after surgery for those not receiving adjuvant therapy. To have a valid PDT timepoint, a blood sample had to be collected within 6 months (180 days) of the end of definitive therapy.

All blood samples were processed by the University of Pittsburgh Biospecimen Core within 14 days of collection. Blood components were separated by centrifugation at 3200 rpm for 15 min. Plasma was stored at −80°C in cryovials in 1.8 mL aliquots, and buffy coat in 0.5 mL aliquots. All study participants were followed for clinical and survival outcomes. Additional data collected included patient demographics, tumor information, lymph node involvement, treatments received, biomarkers identified from blood draws (CEA), and immunohistochemical testing for mismatch repair (MMR) status performed as part of clinical management. Timing of blood draws and CT imaging generally followed the recommended surveillance strategy for patients with stage III CRC [17]. All data were stored in a study-specific REDCap database hosted at the University of Pittsburgh [18, 19].

Formalin-fixed, paraffin-embedded (FFPE) tumor blocks and buffy coat were used to produce each patient-specific MRD assay. H&E-stained tumor sections were evaluated by a pathologist, microdissection was performed to enrich tumor content (> 20% tumor cells), and DNA was extracted from the tumor and from the buffy coat.

### 2.3 | Tumor-Informed MRD Assay

The tumor-informed MRD assay involves two phases: variant discovery and ctDNA detection; see Supporting Information S1: Section S1 for details. Briefly, variant discovery uses whole-exome sequencing (WES) of paired tumor-normal samples from each patient, which was previously validated for the OncoExTra comprehensive genomic profiling assay [20], to identify 50–200 somatic variants specific to the patient’s tumor. The assay filters out variants known to occur in clonal hematopoiesis of indeterminate potential (CHIP) [21, 22]. CHIP-related variants were obtained from the literature [23, 24]. For ctDNA detection, a hybrid capture methodology is employed, which utilizes short DNA sequences (probes) that span, and can thus pair with, the target DNA regions containing the patient-specific variants identified during discovery, to enrich these regions for DNA sequencing. A library containing 30–60 ng of DNA constructed from the cell-free DNA (cfDNA) in each patient’s plasma samples is hybridized to the patient-specific probes and then sequenced. A proprietary algorithm that utilizes the sequencing-derived variant information is used to generate a ctDNA score for each sample and then determine whether ctDNA is present (ctDNA positive) or absent (ctDNA negative) in the plasma samples. A plasma sample is classified as ctDNA positive if either the ctDNA score is above a specific threshold, or if two or more individual variants exhibit a sufficiently high probability of cancer. If neither of these criteria are met, the plasma sample is classified as ctDNA negative. For development of the algorithm, a set of plasma samples from normal donors (*N* = 58) and patients with CRC (*N* = 97), none of which overlap with α-CORRECT samples, was used and the target sensitivity was set to > 95% (see Supporting Information S1: Section S1B). The analytic limit of detection for the MRD assay was determined to be 0.005% tumor fraction. Additional information and analytic validation results are presented in the Supporting Information S1: Section S1. All assays were run in the Exact Sciences’ CLIA-certified, CAP-accredited laboratory and all laboratory personnel were blinded to clinical outcomes.

### 2.4. | Statistical Analyses

Recurrence-free survival (RFS) was defined as the elapsed time from surgical resection to first clinical recurrence (locoregional or distant), or death due to CRC. Subjects were censored at last follow-up or at non-CRC-related death. For the primary analysis, a Cox proportional hazards regression model was used with ctDNA status (positive, negative) during the surveillance period included as a single, time-dependent covariate, where every surveillance ctDNA result for each patient is included. A hazard ratio (HR) with 95% Wald confidence intervals (CI) was calculated and statistical significance was assessed at the 0.05 level. The primary analysis included all eligible patients for whom an MRD assay result was available at one or more prerecurrence surveillance timepoints. Kaplan-Meier methods were used to illustrate the association of ctDNA status with RFS using a simplified, time-invariant categorization of patients to compare two groups, those with ≥ 1 positive result during the surveillance period versus those with negative results only.

The sensitivity of the MRD assay during surveillance was estimated as the proportion of patients who experienced a clinical recurrence who had at least one positive surveillance ctDNA result before recurrence. The specificity was estimated as the proportion of patients who did not recur who never had a positive surveillance ctDNA result. The Wilson score method was used to calculate 95% CIs for the estimates of sensitivity and specificity [25].

Clinical recurrence can occur months after ctDNA positivity (e.g., [13]). This risks incorrectly assigning a positive ctDNA result as a false positive if insufficient time has elapsed following testing to observe a recurrence. To minimize this risk, we decided, a priori, that patients who had a positive ctDNA result with less than 1 year follow-up after their first positive test and whose cancer had not recurred would be excluded from the calculation of specificity. In addition, to minimize incorrectly scoring a result as a false negative, patients who recurred without a ctDNA result in the preceding 215 days were censored 215 days after their last ctDNA result for surveillance analyses. The 215-day cutoff was chosen based on the target frequency of blood draws: it represents just over 7 months, implying two missed blood draws in the first 3 years or a single missed blood draw in the last 2 years of follow up. We also performed analyses using Cox regression and Kaplan- Meier methods for the PS and PDT timepoints to determine their association with RFS. Cox models for these timepoints included adjustment for delayed entry into the risk set as patients could not be included in the analysis before the corresponding ctDNA result.

Several additional analyses were performed. Univariable Cox models for association with RFS were analyzed for the following clinicopathologic variables: CEA status (defined as normal, < 2.5 ng/mL for nonsmokers or < 5.0 ng/mL for smokers, or abnormal, ≥ 2.5 ng/mL for nonsmokers or ≥ 5.0 ng/mL for smokers), the Oncotype DX Colon Recurrence Score assay measured on tumor tissue [26–28], age, American Joint Committee on Cancer (AJCC) category [29], pathologic T (pT) category, pathologic N (pN) category, and mismatch repair (MMR) status. Multivariable models including CEA, Recurrence Score (RS) result, and any variable that was statistically significant in the univariable analyses (i.e., showed an HR statistically significantly different from 1 at the 0.05 level) were used to assess the independent association of the ctDNA result for surveillance, PS and PDT timepoints with RFS. Analysis of each timepoint was performed in separate models. Since CEA status has been previously shown to be associated with recurrence in stage III CRC [30], we also examined the association between CEA and ctDNA status using contingency tables.

Nonparametric interval censoring methods [31] were used to estimate the time from the first positive surveillance ctDNA result to clinical recurrence. Kaplan-Meier methods were also used to compare RFS between patients receiving chemotherapy who were ctDNA positive at the PS timepoint and remained positive at the PDT timepoint versus those who were ctDNA positive at the PS timepoint but became negative at the PDT timepoint, indicating ctDNA clearance following adjuvant chemotherapy.

## 3 | Results

### 3.1 | MRD Assay and Study Cohort

WES identified 172 (range 50–200) variants per patient on average. Of the 134 patients with sufficient tumor available, an MRD assay was produced for 133 (one failed at the library preparation step). A total of 1315 blood samples were available from the 133 patients with an MRD assay. On average, patients had 10 (standard deviation 4.5, range 1–17) blood draws. Circulating tumor DNA results were obtained for 1297 samples (99%). Among these samples, 1029 (79%) yielded ≥ 30 ng of cfDNA, 185 (14%) yielded 20–30 ng of cfDNA, and 83 (6%) yielded < 20 ng of cfDNA. Samples with cfDNA < 30 ng were excluded from analyses as this was the minimum input level used during analytical validation. Samples that failed to meet DNA input requirements often came from the same patient.

Nine patients were not included in any analysis due to insufficient cfDNA in all their plasma samples, assay failure, or being outside all of the pre-specified timepoint analysis windows, leaving 124 patients who were included in at least one analysis (Figure 1B). Of these patients, 47% were female and most had colon cancer (95%; 5% rectal cancer), were over 50 years of age (91%), were non-Hispanic White (94%) and had surgery followed by adjuvant chemotherapy (96%), with the majority (92%) receiving platinum-based chemotherapy (Table 1). Median follow-up time after surgery was 4.8 years and there were 27 recurrences in total. Swimmer plots with assay results and timing of recurrence (if applicable) for the 124 patients are shown in the Supporting Information S1: Section S2. The number of patients with assay results at PS, PDT and surveillance timepoints was 88, 97 and 111, respectively (Figure 1B). Patients were absent from particular timepoints for a variety of reasons including no plasma sample available within the time window, insufficient cfDNA yield (< 30 ng), lack of follow up information, or death/recurrence before the timepoint. For patients included in the analyses, the median number of days after surgery for the PS timepoint was 42 (range: 23–83), and the median number of days after the end of definitive therapy for the PDT timepoint was 43 (range: 0–179).

### 3.2 | Circulating Tumor DNA Detection and RFS

**FIGURE 2 |:**
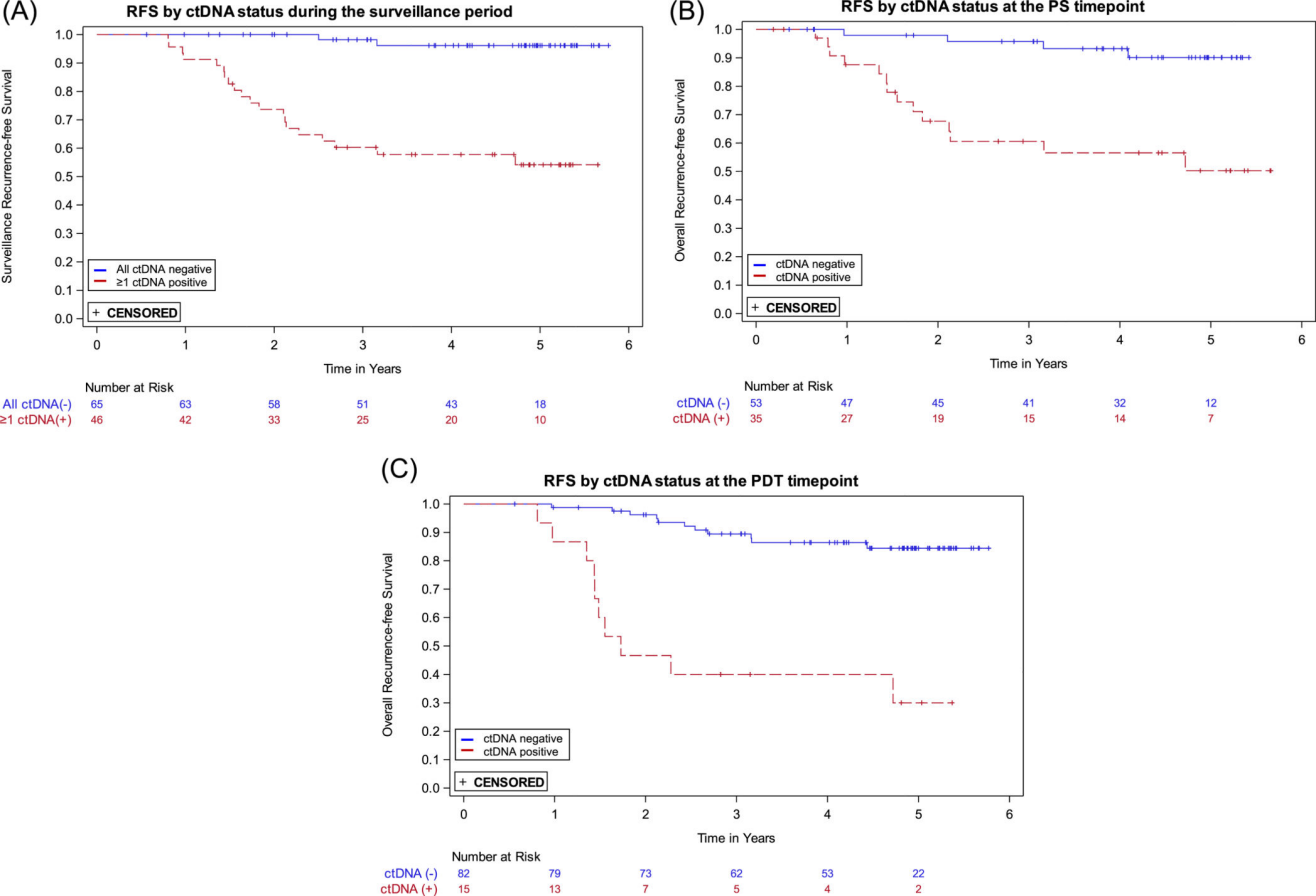
Kaplan-Meier recurrence-free survival (RFS) curves by ctDNA status. (A) At least one positive ctDNA result versus all negative ctDNA results during the surveillance time period. (B) Positive versus negative ctDNA at the postsurgical (PS) timepoint. (C) Positive versus negative ctDNA at the post-definitive therapy (PDT) timepoint.

Plasma ctDNA status was strongly associated with the risk of recurrence. The Cox proportional hazards regression model with ctDNA status as a single, time-dependent variable where each ctDNA result is included during follow up demonstrated that the recurrence HR for a positive versus negative ctDNA result during the surveillance time period was 29.4 (95% CI: 9.9–87.8; *p* < 0.0001) (Table 2). To illustrate the association between ctDNA positivity and RFS, Kaplan-Meier curves for patients with ≥ 1 ctDNA-positive result versus those with all negative results during surveillance are presented in Figure 2A.

The recurrence HRs for plasma ctDNA status, positive versus negative, at the PS and the PDT timepoints were 7.0 (95% CI: 2.3–21.4) and 8.1 (95% CI: 3.4–19.2), respectively (Table 2); the Kaplan-Meier curves are shown in Figures 2B,C. The 3-year RFS estimates for ctDNA positive and negative participants were, respectively, 60.6% and 95.7% for PS and 40.0% and 89.4% for PDT (Table 2).

The sensitivity of the assay for the surveillance period was 90.9% (20 of 22 recurrences were predated by a positive ctDNA result; 95% CI: 72.2%–97.5%) and the specificity was 73.3% (63 of 86 patients who did not recur never had a ctDNA positive result; 95% CI: 63.1%–81.5%). As prespecified in the analysis plan, three patients who had < 1 year of follow-up following their first surveillance ctDNA positive result and did not recur were excluded from the specificity calculation. Three patients who recurred were censored 215 days after their last ctDNA result due to the lack of a result within 215 days prerecurrence. At the PS timepoint, sensitivity and specificity were 77.8% (95% CI: 54.8%–91.0%) and 74.2% (95% CI: 62.6%–83.3%), respectively. At the PDT timepoint, sensitivity and specificity were 47.6% (95% CI: 28.3%–67.6%) and 93.4% (95% CI: 85.5%–97.2%), respectively (Table 2).

### 3.3 | Adjusted Criteria for Assigning ctDNA Status

**FIGURE 3 |:**
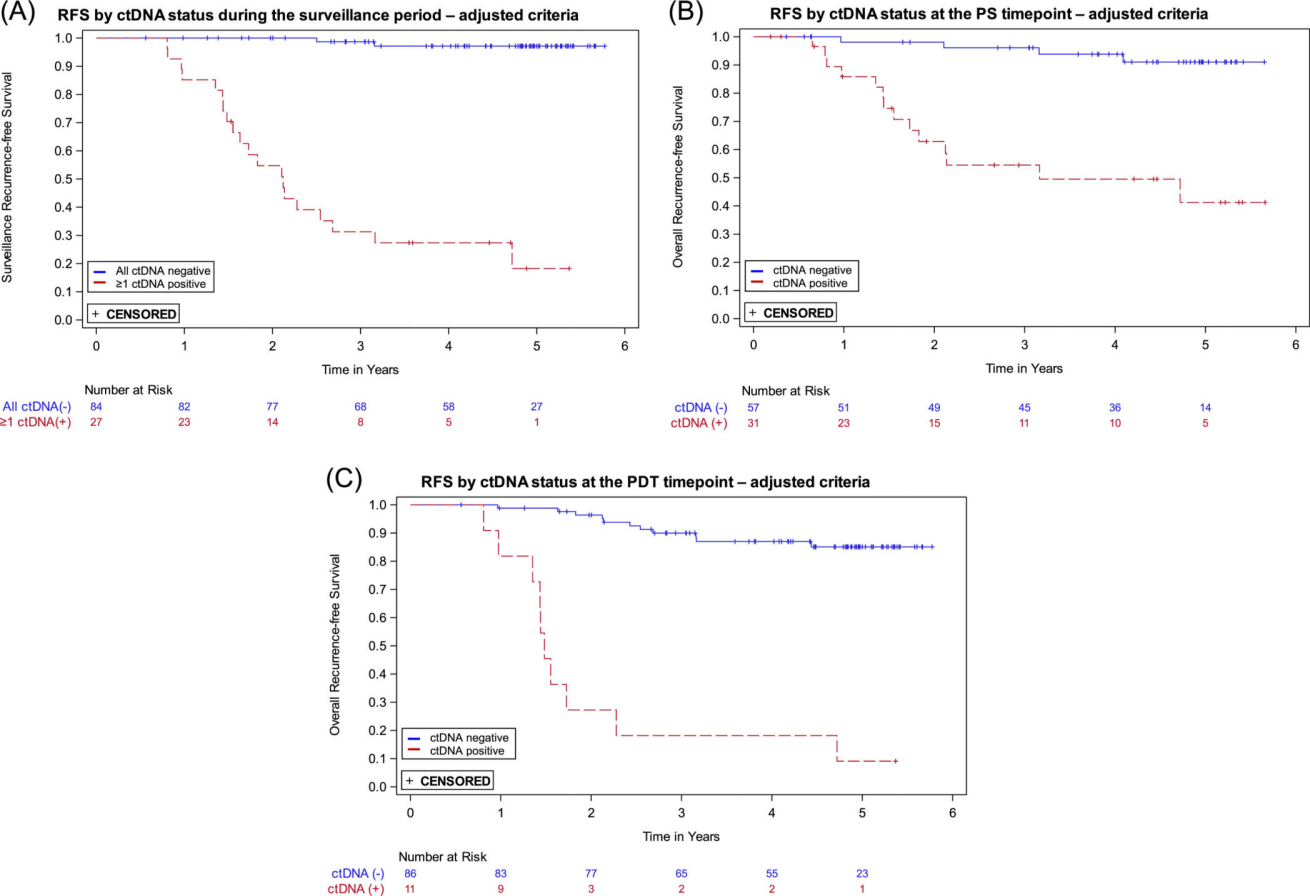
Kaplan-Meier recurrence-free survival (RFS) curves by ctDNA status using the adjusted criteria for ctDNA status determination. (A) At least one positive ctDNA result versus all negative ctDNA results during the surveillance time period. (B) Positive versus negative ctDNA at the postsurgical (PS) timepoint. (C) Positive versus negative ctDNA at the post-definitive therapy (PDT) timepoint.

The MRD assay was developed with a target specificity of > 95%. In the pre-specified analyses presented above, the specificity for ctDNA at the PDT timepoint was 93.4%, and in the surveillance period, which includes the PDT timepoint, the specificity was 73.3%. The lower specificity during the surveillance period is due in part to the evaluation of multiple samples for each patient, which increases the likelihood of obtaining at least one false positive ctDNA result. To increase surveillance specificity, we utilized the independent development sample set and an updated target specificity of 99.5% to adjust the positivity criteria used for ctDNA status determination (see Supporting Information S1: Section S1B for details). The positivity criteria adjustment included two changes: an increase in the ctDNA score threshold, and removal of the two high probability individual variants criterion. With the adjusted criteria, we re-evaluated the ctDNA status of each sample and repeated the prespecified analyses. In the surveillance period sensitivity was unchanged, while specificity improved to 94.3%. Modest improvements in specificity were seen for the PS and PDT timepoints (see Table 2). Recurrence HRs (ctDNA positive vs. negative) increased for all timepoints (see Table 2); Kaplan-Meier curves are shown in Figure 3. All additional analyses described below were performed using ctDNA status determined with the adjusted criteria.

### 3.4 | Additional Analyses

Table 3A shows the univariable Cox model results for ctDNA status, CEA status, RS, age, AJCC stage, pT category, pN category, and MMR status with RFS. The following variables were significantly associated with RFS: ctDNA status at surveillance, PS and PDT timepoints; CEA status at surveillance and PDT timepoints; pT category; and RS. The multivariable results, which include ctDNA status, CEA, RS, and pT category, are shown in Table 3B. Circulating tumor DNA status for the surveillance period was the only variable that remained a significant predictor of RFS (HR: 39.9; 95% CI: 12.0–132.7; *p* < 0.0001). At the PS timepoint, only ctDNA status (HR: 8.7; 95% CI: 2.7–27.8; *p* = 0.0003) remained significantly associated with RFS, and at the PDT timepoint ctDNA status (HR: 24.7; 95% CI: 8.6–70.5; *p* < 0.0001) and pathological T category (HR: 3.2; 95% CI: 1.2–8.4; *p* = 0.0207) remained significantly associated with RFS.

The estimated median lead time between a positive ctDNA result and a clinical diagnosis of recurrence in the surveillance period using interval censoring methods was 10.4 months (95% CI: 5.2–10.7).

**FIGURE 4 |:**
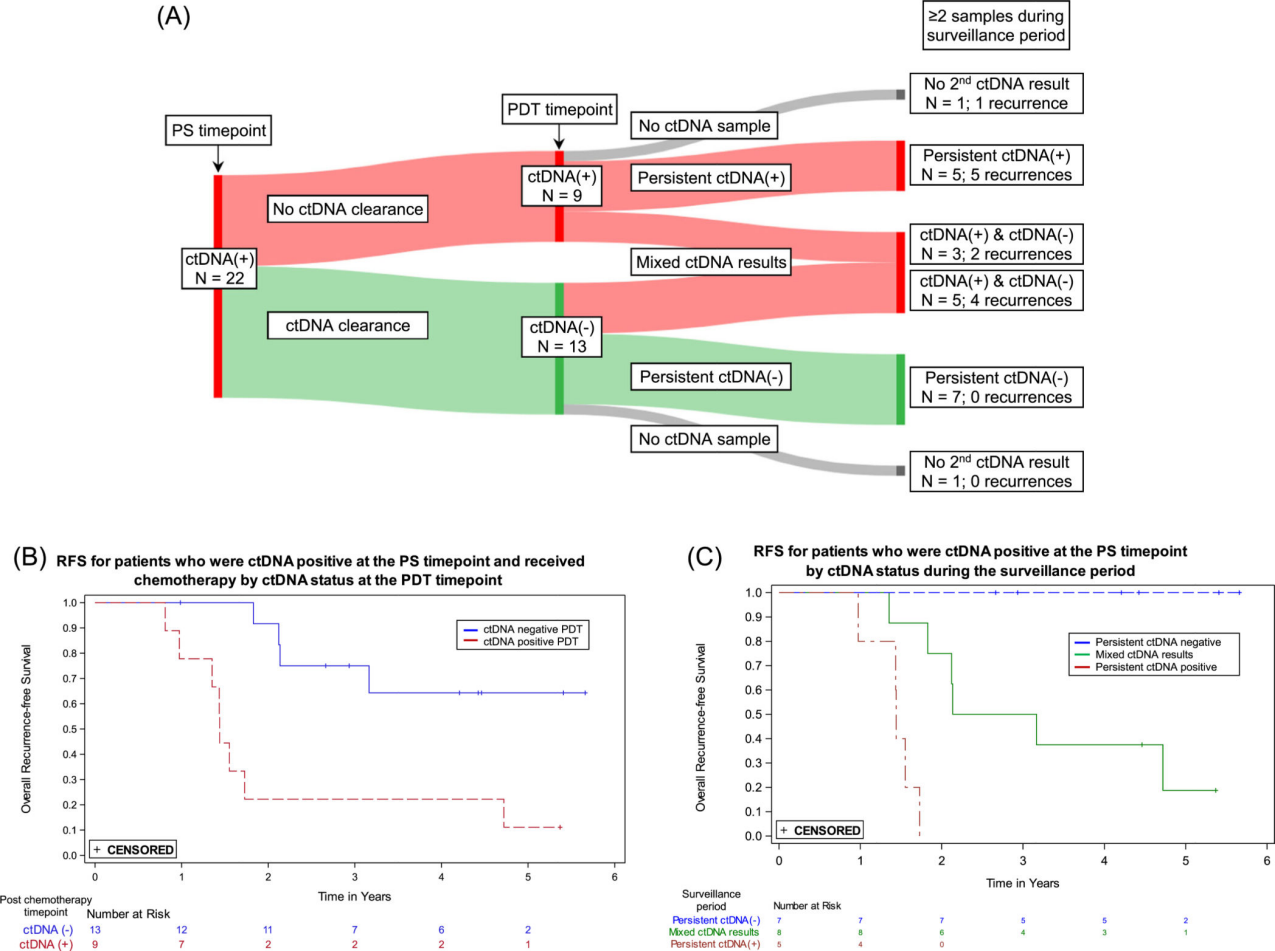
Clearance of ctDNA and recurrences following adjuvant chemotherapy. (A) Sankey plot showing the 22 patients who were ctDNA positive at the postsurgical (PS) timepoint and had a post-definitive therapy (PDT) timepoint ctDNA result. Thirteen of these patients showed clearance, defined as being ctDNA positive at the PS timepoint and ctDNA negative at the PDT timepoint, and nine did not show clearance. The ctDNA results following the PDT timepoint were either consistently positive, consistently negative, mixed–having both positive and negative results during the surveillance period, or absent (no ctDNA result). (B) Kaplan-Meier curves for recurrence-free survival (RFS) by ctDNA clearance status: positive at PS and PDT (no clearance) versus positive at PS and negative at PDT (clearance). (C) Kaplan-Meier curves for RFS for patients who were ctDNA positive at the PS timepoint, had a PDT timepoint sample and at least one additional sample after the PDT timepoint, by ctDNA status during surveillance: persistent ctDNA positive, persistent ctDNA negative, or mixed ctDNA results.

Figure 4A shows the ctDNA status and recurrences for patients who were ctDNA positive at the PS timepoint, received adjuvant therapy, and had an evaluable sample at the PDT timepoint (*N* = 22). Four of the 13 patients who cleared ctDNA between the PS and PDT timepoints recurred, compared to 8 of the 9 patients who were ctDNA positive at both timepoints. For patients who were ctDNA positive at the PS timepoint, RFS was significantly worse for those who remained ctDNA positive versus those who showed ctDNA clearance after receiving adjuvant therapy (HR = 5.6, 95% CI: 1.6–19.3) (Figure 4B). Among the ctDNA positive patients at the PS timepoint, all five who remained positive throughout surveillance recurred, while none of the seven patients who were ctDNA negative at the PDT timepoint and remained negative throughout surveillance recurred (Figure 4A,C).

In an exploratory analysis, we examined the relationship between ctDNA status and recurrence site. The most common sites of recurrence were metastases to the liver or lung, or both (16 of 27 recurrences). Liver metastases appeared to be more likely to show ctDNA positivity than lung metastases (see Supporting Information S1: Section S3).

## 4 | Discussion

Identification of patients with CRC who are at a higher risk of recurrence and earlier identification of impending recurrence may allow for implementation of additional or alternative curative treatments. In this retro-prospective study of 124 stage III CRC patients followed for a median of 4.8 years, we used an analytically validated tumor-informed MRD assay to examine the association between ctDNA status and RFS. The assay interrogates 50 to 200 patient-specific variants, more than most previous assays have pursued, and has an analytical lower limit of detection of 0.005%. We found that ctDNA status is strongly associated with CRC recurrence using both the original and adjusted assay positivity criteria.

With the adjusted criteria, we observed a strong, significant association between ctDNA status at the PS timepoint and risk of recurrence (HR 9.6; 95% CI: 3.2–29.5), which may be relevant for clinical treatment decision making. At the PS timepoint, despite most receiving adjuvant, oxaliplatin-based chemotherapy, ctDNA positive patients had an estimated 3-year RFS of 54.5% compared to 96.1% for those who were ctDNA negative. The sensitivity of the assay at this timepoint was 77.8%; a positive ctDNA result was observed in 14 of the 18 who recurred.

At the PDT timepoint, a negative ctDNA result was highly predictive of a favorable long-term outcome, with a 90.0% RFS at 3 years and assay specificity of 98.7%. In contrast, a positive ctDNA at the PDT timepoint was strongly associated with recurrence (HR 16.7; 95% CI: 6.9–40.3), with a 3-year RFS of only 18.2%. The sensitivity of the assay at this timepoint was only 47.6%, meaning that for a majority of patients who recurred, ctDNA was not detected by the assay in the plasma sample collected after definitive treatment. Several of those who recurred had a positive test shortly thereafter (see swimmer plots, Supporting Information S1: Section S2), suggesting that sampling too soon after completing adjuvant therapy may be misleading regarding outcome. However, recurrence after the PDT timepoint did not develop until many months later in some patients, pointing to the need for sampling during the surveillance period to monitor outcome.

The strongest association between ctDNA status and recurrence was during surveillance (HR 49.6; 95% CI: 16.6–148.3; *p* < 0.0001). Patients who were negative throughout the surveillance period had very low recurrence rates (Figure 3A); only 2 of 84 persistently ctDNA negative patients recurred. Among patients who were ctDNA positive after surgery but cleared ctDNA following adjuvant chemotherapy, all of those who remained ctDNA negative during surveillance did not recur (7/7, Figure 4A), an outcome consistent with previous studies [9]. Of the 13 patients who cleared ctDNA with adjuvant therapy in our study, 69% (9/13) did not recur, consistent with what is reported in the GALAXY trial (82%), taking into account their shorter follow up time of 18 months [12]. In contrast, for the 22 patients who recurred, a positive ctDNA in the surveillance period was observed in 20, for a sensitivity of 90.9%. Eight of the nine patients who were ctDNA positive at both PS and PDT timepoints recurred, consistent with other studies [9]. In our study, ctDNA positivity preceded clinical recurrence by over 10 months, similar to what has been reported in other tumor-informed MRD studies [13, 32]. The results during surveillance are prognostically relevant and could inform adjustments in patient management and treatment strategies, though clinical trials based on ctDNA findings are required to determine clinical utility.

The presence of ctDNA has been shown in multiple studies to be more strongly associated with recurrence risk compared to standard prognostic histopathologic factors and CEA levels [15]. In the present study, ctDNA status remained highly significantly associated with RFS in all multivariable models, underlining its prognostic value for recurrence compared to other factors. The current standard of care for CRC patients after surgery includes CEA level determination, measured every 3–6 months for up to 5 years [33]. CEA status was associated with ctDNA status; abnormal CEA levels occurred more frequently in ctDNA positive than in ctDNA negative plasma samples (see Supporting Information S1: Section S4). Although we observed that CEA status was significantly associated with RFS in a univariable model, it did not remain significant in any of the multivariable models and CEA status did not provide additional prognostic value compared to ctDNA (Table 3).

Clinical trials are investigating the clinical utility of ctDNA status at various decision timepoints. In the VEGA trial (jRCT1031200006), patients with a negative postsurgical ctDNA result are randomized to chemotherapy versus no chemotherapy to investigate therapy de-escalation [34]. In the AL-TAIR trial (NCT04457297), patients with a ctDNA positive result after chemotherapy are randomized to placebo versus trifluridine/tipiracil hydrochloride to investigate therapy escalation [34]. In the CIRCULATE US trial (NCT05174169), ctDNA status is used to determine intensification or deintensification of therapy: ctDNA positivity post-surgery or during surveillance leads to randomization to 6 months of FOLFOX/CAPOX versus FOLFIRINOX, while ctDNA negativity leads to randomization between no chemotherapy (with continued surveillance) or FOLFOX/CAPOX chemotherapy [35]. In the IMPROVE-IT2 trial (NCT 04084249), a positive surveillance ctDNA result triggers radiological assessment to detect possible recurrence [36]. These trials will give important insights into the clinical utility of ctDNA status at various timepoints and under different clinical circumstances.

Strengths of this study include that it is a multicenter, prospective, well-curated cohort of patients with stage III CRC, the majority of whom were treated with platinum-based chemotherapy. The 4.8 year median follow-up period is longer than in most other studies [7–14], and is consistent with the American Society of Clinical Oncology (ASCO) and National Comprehensive Cancer Network (NCCN) recommended follow-up period of 5 years, ideal for testing the association of ctDNA status with recurrence [17, 37]. There is robust serial blood sampling, with an average of 10 blood draws per patient. The MRD assay, with an analytical limit of detection of 0.005% tumor fraction and algorithm criteria for ctDNA status determination developed in an independent data set, utilizes 50-200 patient-specific variants identified using WES, with germline subtraction and exclusion of CHIP variants for detection of ctDNA.

Limitations of the study include that it was unable to evaluate whether adjuvant chemotherapy is beneficial given that the design was observational. The sample size was modest, and some plasma samples yielded insufficient cfDNA, leading to loss of patients for some analyses. The technical success of the cfDNA extraction was 79% for > 30 ng DNA, the pre-specified threshold for analysis. However, the success rate increased to 93% in exploratory analyses that included samples with > 20 ng to ≤ 30 ng DNA, which were equally informative for outcome results (see Supporting Information S1: Section S5). Some patients were excluded from PS and PDT analyses due to blood collection occurring outside of the prespecified time windows. Relaxing the time period requirements, such that these patients could be included in the analyses, gave similar results (see Supporting Information S1: Section S5). Finally, we adjusted the criteria for ctDNA status determination during the study utilizing an independent sample set by increasing the target specificity (from 95% to 99.5%) to reduce the number of false positives. The adjusted criteria will be confirmed and extended in a prospective, independent study of > 400 patients with stage II, III, and IV CRC (β-CORRECT).

## 5 | Conclusion

In conclusion, ctDNA status determined by our patient-specific, tumor-informed ctDNA assay was strongly prognostic for risk of recurrence in patients with stage III colorectal cancer at all timepoints. The prognostic association of ctDNA status with recurrence was greater than other clinical factors, including CEA, which is the current standard of care.

## Supplementary Material

diergaarde supporting info

Additional supporting information can be found online in the Supporting Information section.

## Figures and Tables

**TABLE 1 |:** Characteristics of the 124 patients included in at least one analysis.

Characteristic	Value	Characteristic	Value

Site of cancer, *n* (%)		Histopathologic grade, *n* (%)	
Colon	118 (95.2)	Low	26 (21.0)
Rectum	6 (4.8)	Mod. Differentiated	67 (54.0)
		High	26 (21.0)
Age at diagnosis in years		Other/NA	5 (4.0)
Mean (SD)	64.5 (10.8)		
		Pathologic T category, *n* (%)	
Age Range in years, *n* (%)		pT1	3 (2.4)
< 50	11 (8.9)	pT2	15 (12.1)
50–64	49 (39.5)	pT3	74 (59.7)
65+	64 (51.6)	pT4a	28 (22.6)
		pT4b	4 (3.2)
Gender, *n* (%)			
Male	66 (53.2)	Pathologic *N* category, *n* (%)	
Female	58 (46.8)	pN1	74 (59.7)
		pN2	50 (40.3)
Ethnicity, *n* (%)			
Not Hispanic or Latino	124 (100.0)	Neoadjuvant therapy received, *n* (%)	
		No	122 (98.4)
Race, *n* (%)		Yes	2 (1.6)
White	116 (93.5)		
Black	2 (1.6)	MMR status, *n* (%)	
Other	6 (4.8)	Proficient	98 (79.0)
		Deficient	19 (15.3)
Smoking status, *n* (%)		Unknown	7 (5.6)
Current	13 (10.5)		
Former	51 (41.1)	Adjuvant therapy received, *n* (%)	
Never	60 (48.4)	No	5 (4.0)
		Yes	119 (96.0)
Stage AJCC 8th edition*, *n* (%)			
IIIA	16 (12.9)	Adjuvant chemotherapy type, *n* (%)	
IIIB	76 (61.3)	5FU	9 (7.6)
IIIC	32 (25.8)	5FU + Ox	110 (92.4)
Histopathologic type, *n* (%) Adenocarcinoma	110 (88.7)	Follow up time after surgery in years Median (min–max)	4.8 (0.2–5.8)
Medullary carcinoma	1 (0.8)		
Mucinous adenocarcinoma	12 (9.7)		
Signet-ring cell	1 (0.8)		

Abbreviations: 5FU, fluorouracil; MMR, mismatch repair; Ox, oxaliplatin; SD, standard deviation.

* American Joint Committee on Cancer staging 8th edition [29].

**TABLE 2 |:** Hazard ratios (HR), sensitivity, and specificity, with 95% confidence intervals (95% CI), for the association of ctDNA status with recurrence-free survival (RFS) at the surveillance, postsurgical (PS), and post-definitive therapy (PDT) timepoints. Three-year RFS estimates for the PS and PDT timepoints are also shown. Results are given for ctDNA status determined both with the original criteria and with the adjusted criteria.

Timepoint	Statistic	Original criteria	Adjusted criteria

Surveillance (*N* = 111)	HR (95% CI)	29.4** (9.9–87.8)	49.6** (16.6–148.3)
	Sensitivity (95% CI)	20/22 = 90.9% (72.2%–97.5%)	20/22 = 90.9% (72.2%–97.5%)
	Specificity (95% CI)	63/86 = 73.3% (63.1%–81.5%)	82/87 = 94.3% (87.2%–97.5%)
PS (*N* = 88)	HR (95% CI)	7.0* (2.3–21.4)	9.6** (3.2–29.5)
	Sensitivity (95% CI)	14/18 = 77.8% (54.8%–91.0%)	14/18 = 77.8% (54.8%–91.0%)
	Specificity (95% CI)	49/66 = 74.2% (62.6%–83.3%)	53/66 = 80.3% (69.2%–88.1%)
	RFS at 3 yrs ctDNA(+), ctDNA(−)	60.6%, 95.7%	54.5%, 96.1%
PDT (*N* = 97)	HR (95% CI)	8.1** (3.4–19.2)	16.7** (6.9–40.3)
	Sensitivity (95% CI)	10/21 = 47.6% (28.3%–67.6%)	10/21 = 47.6% (28.3%–67.6%)
	Specificity (95% CI)	71/76 = 93.4% (85.5%–97.2%)	75/76 = 98.7% (92.9%–99.8%)
	RFS at 3 years ctDNA (+), ctDNA (−)	40.0%, 89.4%	18.2%, 90.0%

* *p* < 0.001,

** 2*p* < 0.0001.

**TABLE 3 |:** Univariable (A) and multivariable (B) Cox model results for associations with recurrence-free survival.

A						

Variable	HR compares		Sample sizes		HR (95% CI)	*p* value

Surveillance ctDNA status^a^	Positive versus negative		111		49.6 (16.6–148.3)	**< 0.0001**
Surveillance CEA status^a^	Abnormal^b^ versus normal		114		9.2 (4.0–20.9)	**< 0.0001**
PS ctDNA status	Positive versus negative		31/57		9.6 (3.2–29.5)	**< 0.0001**
PS CEA status	Abnormal versus normal		7/114		1.8 (0.4–7.8)	0.4050
PDT ctDNA status	Positive versus negative		11/86		16.7 (6.9–40.3)	**< 0.0001**
PDT CEA status	Abnormal versus normal		13/97		2.7 (1.0–7.2)	**0.0481**
RS	10-unit increase		110		1.5 (1.0–2.2)	**0.0355**
AJCC stage	IIIC versus IIIA/B		32/92		1.6 (0.7–3.5)	0.2792
pT category	pT4 versus pT1-3		32/92		3.0 (1.4–6.4)	**0.0048**
pN category	pN2 versus pN1		50/74		1.1 (0.5–2.4)	0.8217
MMR status	Deficient versus proficient		19/98		0.7 (0.2–2.4)	0.5742
Age at diagnosis	10-year increase		124		1.0 (0.7–1.5)	0.9078

B						

	Surveillance ctDNA status (time-dependent) (*N* = 99)		PS ctDNA status (*N* = 78)		PDT ctDNA status (*N* = 86)	
Variable	HR (95% CI)	*p* value	HR (95% CI)	*p* value	HR (95% CI)	*p* value

ctDNA status	39.9 (12.0–132.7)	**< 0.0001**	8.7 (2.7–27.8)	**0.00013**	24.7 (8.6–70.5)	**< 0.0001**
CEA status^c^	1.5 (0.5–4.2)	0.4300	0.5 (0.1–3.9)	0.4843	—	—
RS	1.1 (0.7–1.8)	0.5727	1.5 (0.9–2.6)	0.1233	1.2 (0.7–2.0)	0.5905
pT category	2.2 (0.9–5.7)	0.0971	2.0 (0.7–5.7)	0.1968	3.2 (1.2–8.4)	**0.0207**

Abbreviations: AJCC, American joint Committee on Cancer; CEA, carcinoembryonic antigen; MMR, mismatch repair; PDT, postdefinitive therapy; pN, pathological node; PS, postsurgical; pT, pathological tumor; RS, Oncotype DX Colon Recurrence Score test.

a Time-dependent variable (multiple ctDNA results per patient).

b Abnormal is ≥ 2.5 ng/mL for nonsmokers, ≥ 5 ng/mL for smokers.

c In the multivariable model including the PDT ctDNA timepoint, CEA was not significant (*p* = 0.3073) but was removed due to the small number of CEA and ctDNA positive results, which caused instability in the parameter estimates.

## Data Availability

Most of the data supporting the findings of this study are available within the manuscript and supporting information. Individual patient data can be requested from the corresponding author.
