# Supplementary material for: Circulating Tumor DNA as a Marker of Recurrence Risk in Stage III Colorectal Cancer: The α-CORRECT Study
Source: J Surg Oncol. Author manuscript; Available in PMC 2025 Jul 28. (PMC12302971; doi:10.1002/jso.27989)
Supplement: Supplementary file 1 [file NIHMS2089598-supplement-diergaarde_supporting_info.docx]

Supplementary Materials for Diergaarde et al.

S1. Overview and analytical validation of the MRD assay.

A. Assay Overview.
B. Algorithm Development and Criteria Adjustment.
C. Summary of Analytical Validation Results.
D. Samples and Methods.
E. Limit of Detection (Analytical Sensitivity).
F. Precision.
G. Method Comparison.
H. Analytical Specificity.

S2. Swimmer plots for the 124 patients in the α-CORRECT study.

S3. Recurrence location and ctDNA positivity.

S4. Association between ctDNA and CEA.

S5. Results with lower cfDNA input cutoff.

S1. Analytical validation of the MRD assay.

S1A. Assay Overview

The MRD assay is a tumor-informed assay that utilizes whole-exome sequencing (WES) of a patient's tumor and their normal (germline) DNA to identify somatic variants unique to their tumor. A subset of 50-200 somatic variants are then used to determine whether ctDNA is present in their blood. This assay was developed, validated, and is performed by Exact Sciences under CLIA regulations in a laboratory accredited by the College of American Pathologists.

The assay involves two workflows, discovery and detection (Figure S1A.1). The discovery phase requires a formalin-fixed paraffin-embedded (FFPE) tumor tissue sample from biopsy or surgery and a blood specimen for WES. The assay specifically focuses on single nucleotide variants (SNVs) and small insertion/deletions (indels; ≤ 12 bp). WES is followed by subtraction of variants present in the normal sample from variants in the tumor sample, resulting in the identification of tumor-specific (somatic) variants. Variants in regions that have been described in the literature as occurring in clonal hematopoiesis of indeterminate potential (CHIP) are explicitly avoided (Croitoru, Cazacu et al. 2021; Feusier, Arunachalam et al. 2021). The discovery phase of the MRD assay utilizes the same validated wet-lab workflow as the OncoExTra® assay (White, Szelinger et al. 2021). Once somatic variants are identified, they are ranked using various characteristics to maximize sensitivity and specificity during the detection phase. A bespoke hybrid capture panel is then synthesized using the top 50-200 variants (numbers were determined by simulation) on this ranked list.

The detection workflow is a blood-based enrichment assay designed to identify tumor variants using the hybrid capture panel. Thirty to 60ng of patient cfDNA extracted from plasma is enriched by hybridization, and subsequently sequenced to determine presence of the tumor-specific variants. A proprietary bioinformatic algorithm determines whether a sample is scored ctDNA positive (See Supplementary Materials S1B).

Figure S1A.1: Overview of discovery and detection phases of the MRD assay.

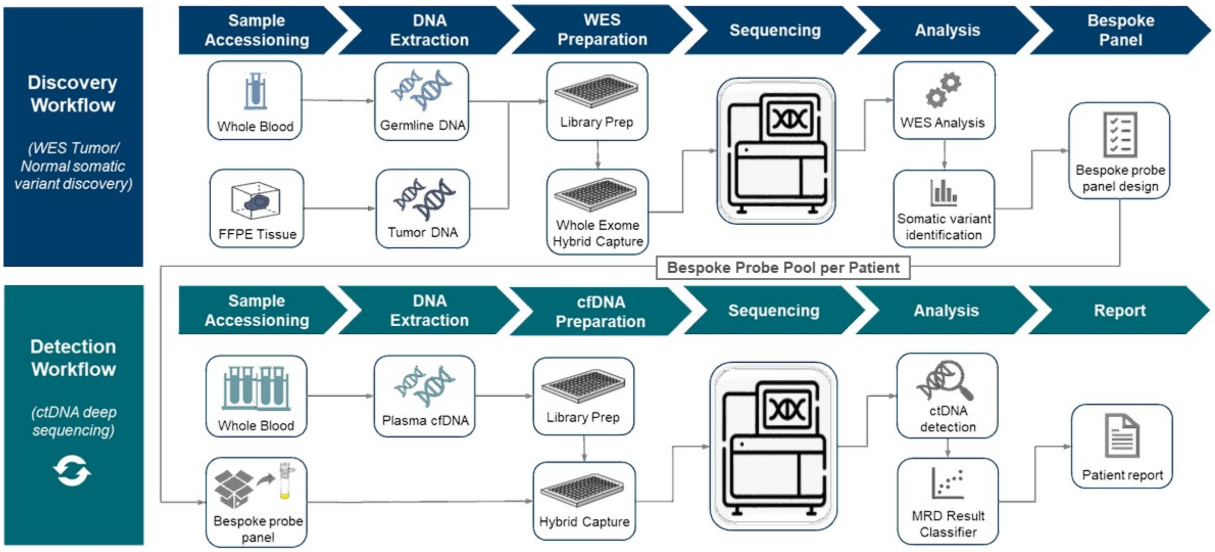

S1B. Algorithm Development and Criteria Adjustment

Algorithm development

To develop the ctDNA positivity scoring algorithm, a ctDNA score was first derived using a set of development samples that were independent of the α-CORRECT clinical samples. A total of 360 replicates were used, including 99 replicates from 97 blood samples from patients with CRC (13 stage I, 42 stage II, 30 stage III, and 12 stage IV) and 261 replicates from 58 normal blood donor samples. Each of the 97 patients with CRC had a personalized MRD assay developed that was then used to test both their own blood sample and some of the 261 normal replicates (each MRD assay was used to test 2.7 normal sample replicates on average). The demographics of the patients with CRC and the normal blood donors are shown in Table S1B.1. The CRC patient population was older and included a higher percentage of females than the normal population.

TableS1B.1. Characteristics of the patient cohorts used to develop the MRD assay algorithm.

Cohort characteristics	Normal	CRC
Total, n	58	97
Female, n (%)	23 (39.7%)	52 (53.6%)
Age (yrs), mean (SD)	50.4 (±14.8)	64.7 (±10.0)
Stage I, n (%)	–	13 (13.4%)
Stage II, n (%)	–	42 (43.3%)
Stage III, n (%)	–	30 (30.9%)
Stage IIV, n (%)	–	12 (12.4%)

Sequencing data for each bespoke probe panel was processed by commercially available variant callers. Variant data were used as predictors in a logistic regression model with cancer status as the response variable. Outcomes from the logistic regression model were used to generate the ctDNA score for each sample. The threshold for ctDNA positivity for the ctDNA score was selected as the 95th percentile of a probability distribution fit to the ctDNA scores from the normal samples. Samples were also considered ctDNA positive if two or more individual variants exhibited a sufficiently high probability of cancer.

Positivity criteria adjustment

When initially developed, the ctDNA positivity criteria were designed to maximize sensitivity while keeping the specificity above 95% for a single test. However, during surveillance, multiple samples are taken from each patient and each tested sample increases the probability a ctDNA negative patient will produce at least one positive result. Consequently, specificity measured across multiple tests is reduced. The initial analyses of the surveillance data from the α-CORRECT study confirmed that: while specificity at the PDT timepoint was 93.4%, the surveillance period specificity was 73.3%.

The development samples (Table S1B.1) were used to adjust ctDNA positivity criteria by eliminating the criterion for two high probability individual variants and increasing the ctDNA score threshold. The new ctDNA score threshold was chosen to yield a serial specificity of 96% across eight independent tests. This corresponds to a single-test specificity of 99.5%. The new threshold was found by fitting a probability distribution to the ctDNA score from the normal samples and selecting the 99.5th percentile. This adjustment caused a reduction in sensitivity in the development sample set from 77.8% to 72.7% but did not alter sensitivity in the α-CORRECT samples.

S1C. Summary of Analytical Validation Results

An overview of the MRD assay analytical validation results is provided in Table S1C.1. The analytical validation and the initial data analyses described in the manuscript text were first performed with the original positivity criteria and were later reanalyzed with the adjusted positivity criteria (see Supplementary Materials S1B). Experimental details that produced these results are presented in Supplementary Materials S1D-H below. The analytical validation studies were designed based on Clinical and Laboratory Standards Institute (CLSI) guidelines and BLOODPAC recommendations.

Table S1C.1: Analytical performance attributes established for ctDNA detection with the MRD assay, including limit of detection (LoD) as a measure of analytical sensitivity, precision (reproducibility), analytical specificity, and a method comparison study. For LoD and 1x-6x LoD (0.005-0.030% tumor fraction (TF)) precision experiments, the assay was deployed using a minimum variant set (n=50) and minimum DNA input (30ng). For blank (0.000% TF) precision experiment studies, the assay was deployed using a maximum variant set (n=200) and maximum DNA input (60ng). Top line of each row gives results with original positivity criteria; bottom line gives results with adjusted positivity criteria.

Measurement	Results	Sample Type
LoD	0.005% TF<br>0.005% TF	3 Tumor cell lines (70-139 replicates / cell line) at 5 dilution levels (20-80 replicates / level).
Precision / Reproducibility (percent agreement to majority)	**2x-6x LoD (0.010-0.030% TF):**<br>100% detected (widest 95% CI: 83.9-100%)<br>100% detected (widest 95% CI: 83.9-100%)	3 Tumor cell lines at 4 dilution levels (20-92 replicates / level)
	**1x LoD (0.005% TF):**<br>98.7% detected (95% CI: 93.3-99.9%)<br>98.7% detected (95% CI: 93.3-99.9%)	3 Tumor cell lines at LoD (80 replicates)
	**Wild type (0.000% TF):**<br>96.7% not detected (95% CI: 88.6-99.1%)<br>96.7% not detected (95% CI: 88.6-99.1%)	3 Matched normal cell lines with no tumor cell line DNA (60 replicates)
Analytical Specificity	97.7% (95% CI: 88.0-99.9%)<br>100% (95% CI: 91.8-100%)	11 unique patients, 4 replicate samples / patient (44 replicates total)
Method Comparison[a]	**PPA:** 95.3% (95% CI: 84.5-98.7%)<br>95.3% (95% CI: 84.5-98.7%)<br>**NPA:** 50.0% (95% CI: 33.2-66.8%)<br>63.3% (95% CI: 45.5 -78.1%)<br>**OPA:** 76.7% (95% CI: 65.8-84.9%)<br>82.2% (95% CI: 71.9-89.3%)	53 unique patients, 1-3 timepoint samples per patient (73 samples total)

[a]Comparison to results using Illumina's TruSight Oncology 500 assay to assess diagnostic accuracy.
[b]Values indicated for all samples regardless of TF.
LoD = limit of detection. CI = confidence interval TF = tumor fraction. PPA = positive percent agreement; NPA = negative percent agreement; OPA = overall percent agreement.

S1D. Samples and Methods

Both contrived and clinical samples were used for the analytical validation of the MRD assay. Due to the rarity of matched tumor, normal cell lines, contrived samples were generated using DNA from three stage IIA, grade 3 triple negative breast tumor cell lines (HCC1599, HCC1143, and HCC1187) and their matched normal cell lines (HCC1599BL, HCC1143BL, and HCC1187BL, respectively). All cell lines were purchased from ATCC (www.atcc.org). To create DNA samples with varying TF, mean variant allele frequencies (VAF) for each tumor line were first determined using replicate samples of normal and tumor DNA mixtures (0:100, 95:5, 99:1, and 100:0 respective ratios) and sequenced on two runs with a NextSeq 2000. A linear regression of the measured mean VAF versus expected TF was then used to determine the mixtures needed to generate the desired target TFs.

A selection of clinical samples procured from commercial vendors was also utilized. A set of FFPE tumor samples and both pre- and post-treatment blood samples from 76 randomly chosen patients with colorectal cancer (CRC) were used (Table S1D.1). A total of 73 blood samples from 53 of these patients that passed quality control (Table S1D.2) were tested using both the MRD assay and a comparative method, Illumina's TruSight Oncology 500 ctDNA v1 (herein referred to as TSO500) (Illumina 2024). For confirmation of LoD from cell lines, single blood samples from each of five of these patients that were ctDNA positive by both assays were diluted to achieve target TFs with DNA pooled from 18 prescreened healthy individuals.

The data analysis was first completed with the results from an algorithm that utilized the original positivity criteria, which were later adjusted to address suboptimal specificity observed in the initial analysis of the α-CORRECT data (see Supplementary Materials S1B). Results obtained with both the original and adjusted positivity criteria are presented in Supplementary Materials Table S1C.1 and in S1E-H.

Table S1D.1: Clinical samples from patients with colorectal cancer with unknown ctDNA status used in the method comparison study. Blood samples from a subset of these patients were also used for clinical confirmations of LoD and precision, and in the analytical specificity study (Tabl1S1D.2). A. Patient characteristics of entire cohort. B. Sample counts of 53 patients with samples passing quality control. All patients had one blood sample tested that was drawn before any treatment had been received.

A.

Cohort characteristics	
Total, n	76
Female, n (%)	31 (41%)
Age (yrs), mean (SD)	66 (±12.5)
Stage II, n (%)	25 (33%)
Stage III, n (%)	47 (62%)
Unknown stage, n (%)	4 (5%)
Rectal cancer, n (%)	13 (17%)
Colon cancer, n (%)	63 (83%)

B.

Blood samples	Unique patients (n)	Total Samples (n)
1 timepoint	35	35
2 timepoints	16	32
3 timepoints	2	6
Total	53	73

Table S1D.2: Characteristics of the patients with colorectal cancer whose samples were used in the LoD, precision and analytical specificity validation experiments.

Cohort characteristics	
Total, n	19
Female, n (%)	9 (47%)
Age (yrs), mean (SD)	70.6 (± 9.4)
Stage II, n (%)	9 (47%)
Stage III, n (%)	10 (53%)
Rectal cancer, n (%)	1 (53%)
Colon cancer, n (%)	18 (95%)

S1E. Limit of Detection (LoD) (Analytical Sensitivity)

S1E1. LoD – Contrived Samples

Methods: Contrived DNA samples from three cell lines (see Supplementary Materials S1C above) were used to establish the LoD of the assay, defined as the lowest ctDNA TF for which the MRD assay returned a positive ctDNA result at least 95% of the time (Pierson-Perry, Vaks et al. 2012). Three independent cell-line pairs (tumor + normal) were run through the discovery pipeline to identify variants unique to each pair's tumor cell line. Cell-line specific variant panels were then used to detect ctDNA in the contrived samples. Samples were generated by diluting tumor DNA with matched normal DNA to generate five target TFs, 0.030%, 0.020%, 0.015%, 0.010%, 0.005%, and each was tested for ctDNA positivity using the MRD assay. Following the recommendation of BLOODPAC (Godsey, Silvestro et al. 2020; Baden, Lin et al. 2024), the LoD was established by challenging the assay in two ways, with minimum acceptable probe panel sizes (50 targets) and DNA amounts equivalent in performance to the minimum acceptable cfDNA input into the library (30ng). The LoD was established independently for two reagent lots across the three cell lines, and the maximum value was accepted as the LoD. Each sample was tested 5-20 times per dilution level and reagent lot. Since all tested TF concentrations in the contrived samples met the *a priori* definition of consistent detection of 95% or greater in positive samples, the data was not suitable for probit regression and the LoD was determined to be the lowest TF tested, 0.005%.

Results: A total of 279 out of 280 ctDNA positive samples from the three cell lines passed quality control checkpoints and gave a ctDNA result. Results from the cell lines were not affected by adjustment of the positivity criteria. Of the 279 valid results, 278 (99.6%) resulted in a ctDNA positive finding for both the original and adjusted positivity criteria (Table S1E.1). Combining results over all cell lines and reagent lots, at the lowest ctDNA concentration tested (0.005% TF), 79 of 80 samples had an aggregate ctDNA detection rate of 98.8% (95% CI: 93.3%-99.9%). At higher ctDNA frequencies (≥ 0.01%) all samples had 100% ctDNA positive results.

Conclusions: Using three contrived samples from tumor cell lines, the limit of detection was determined to be 0.005% TF with both the original and adjusted positivity criteria.

Table S1E.1: The frequency of ctDNA positive results in the contrived samples used for determining sensitivity at five different ctDNA tumor fractions (TFs) for limit of detection. Original and adjusted positivity criteria gave identical results.

Sample ID	Target TF (%)	ctDNA+/Total Replicates	Reagent Lot
1187	0.030	10/10	1 and 2
	0.020	10/10	1 and 2
	0.015	10/10	1 and 2
	0.010	39/39[a]	1 and 2
	0.005	39/40	1 and 2
1143	0.030	5/5	1
	0.020	5/5	1
	0.015	20/20	1
	0.010	20/20	1
	0.005	20/20	1
1599	0.030	5/5	2
	0.020	5/5	2
	0.015	20/20	2
	0.010	20/20	2
	0.005	20/20	2

[a]One replicate failed quality control

S1E2. LoD – Clinical Samples

Methods: The clinical samples from five CRC patients, two females and three males, one stage II and four stage III, consisting of both tumor tissue and whole blood, were used to confirm the LoD established by the contrived samples. From each patient, tumor and normal (from blood) sequencing data were used for the MRD assay discovery phase. Blood samples from the same patients were then tested with the assay at three different dilutions that spanned a range of TFs (0.0150%, 0.0075%, 0.0030%), with two replicates per patient and dilution level, for a total of 30 specimens tested. Each of the two replicates within a patient and dilution level was obtained using a different reagent lot. Clinical samples were diluted using 5 different pooled DNA samples made from 6-7 healthy individuals pre-screened against the specific somatic variants of each of the 5 patients to prevent introduction of false signal. To challenge the performance assessment of the assay, the minimum cfDNA library input of 30ng was used for this study. Standard patient-specific probe sets were designed and

used for each patient in this study, containing up to 200 targets per set, as would be used in typical clinical practice with this assay.

Results: The size of the patient-specific probe sets designed for the clinical samples in this study ranged from 145 to 200. Four of the five clinical samples were tested with six replicates per clinical sample, the fifth sample only had enough cfDNA for 5 full assay replicates, resulting in 29 replicates from five original specimens tested; all produced valid results. The clinical samples produced results consistent with the established LoD for both the original and adjusted positivity criteria. The original positivity criteria had 100% ctDNA detected for all 29 results across the TF dilutions, replicates, and reagent lots, while the adjusted positivity criteria gave 100% ctDNA detected for the dilution levels greater than the previously established LoD 0.005% TF in all 5 patient results (Table S1E2.1). With the adjusted positivity criteria, the oldest (87 years) of the 5 patients had no ctDNA detected at the 0.003% TF dilution level (0/2 detected), and the other 4 patients had 100% detection in the 8 replicates also tested at this dilution fraction.

Conclusions: Using tumor cell lines, the LoD for the MRD assay was determined to be 0.005% TF. Results from clinical samples confirmed the LoD established by the cell lines.

Table S1E2.1: LoD in clinical samples that span the 0.005% LoD established using cell lines. Results are combined from five patients diluted to the three levels shown here with two replicates per patient, each replicate using a different reagent lot. TF = tumor fraction.

Target TF (%)	ctDNA+/Total Replicates[a]	ctDNA+/Total Replicates[b]
0.015 (3.0x LoD)	9/9[c]	9/9[c]
0.0075 (1.5x LoD)	10/10	10/10
0.003 (0.6x LoD)	10/10	8/10

[a]MRD assay with original positivity criteria
[b]MRD assay with adjusted positivity criteria
[c]One replicate did not produce a valid result

S1F. Precision

Methods: The reproducibility of the MRD assay (calculated as the percent agreement of repeated testing to the majority test result for a given condition) was tested using both expected positive contrived samples (three tumor cell lines at five dilution levels) and negative contrived samples (three matched normal cell lines) under reproducibility conditions (multiple runs, three instrument sets, three reagent lots and three operator groups). The cell-line specific probe sets designed for the tumor cell lines were also applied to their respective matched normal cell-line samples. All samples were analyzed in the same CAP/CLIA lab.
To confirm the reproducibility of the assay established by the cell line data, three clinical samples spanning the range of the test and pre-screened by the TSO500 assay were also tested repeatedly (one high positive, one low positive and one negative sample). The positive samples were diluted with pooled healthy sample DNA in the same manner as for the clinical samples in the LoD experiments (see Supplementary Materials S1E2) to 6x and 3x LoD, or 0.030% and 0.015% TF, respectively. From these patients, five replicates at each dilution (one dilution level per patient) were tested with the assay under conditions with different reagent lots, operator teams, and instrument sets over three runs.
For both cell line and clinical samples, the assay performance assessment was challenged by running minimum cfDNA inputs for the expected positive samples and maximum cfDNA inputs for the expected negative samples. Cell line results were further challenged by selecting minimum sized cell line-specific probe sets (50 targets each), while clinical samples were tested with the standard bespoke probe set that would be designed for each patient.

Results: Due to consistency of results across all cell lines, percent agreement was calculated in aggregate, within dilution level. At TFs varying from 2x to 6x LoD, or 0.010% to 0.030% TF, with 32 to 92 replicates per dilution level, the MRD assay showed reproducibility in contrived samples of 100.00% positive agreement (widest 95% CI: 83.9%-100%) with both the original and adjusted positivity criteria. At the LoD of 0.005% TF, reproducibility of 98.7% positive agreement (79/80, 95% CI:93.3-99.9%) was observed across all factors tested with both the original and adjusted positivity criteria. Normal cell lines returned 96.7% negative agreement with the original positivity criteria (58/60, 95% CI:88.6-99.1%) and 100% negative agreement with the adjusted positivity criteria (60/60, 95% CI: 94.0-100%) (Table S1F.1A).

Clinical sample results confirmed the precision established by the cell line results. Across three clinical samples obtained from stage II CRC patients, one female and two males, spanning the range of the assay, five replicate tests of each sample showed perfect agreement to the majority and expected result. Results for the clinical samples were the same with both the original and adjusted positivity criteria (Table S1F.1B).

Conclusions: With the original and the adjusted positivity criteria the reproducibility of the MRD assay for contrived samples was 98.75% agreement or better in positive samples at concentrations from the LoD of 0.005% TF to 0.030% TF. Reproducibility in ctDNA negative contrived samples was 96.7% with the original positivity criteria and 100% with the adjusted positivity criteria. Clinical patient samples (n=3 patients, with a total of 15 results) showed precision consistent with both the positive and negative contrived sample results (100% agreement to TSO500 assay pre-screening result for each sample). Clinical sample results were unchanged between the original and adjusted positivity criteria.

Table S1F.1: Precision estimates in contrived cell line (A) and clinical (B) samples. Except for TF=0.000% in contrived samples, both the original and adjusted positivity criteria gave the same percent agreement to majority. For TF=0.000% in contrived samples, results with the original positivity criteria are shown on the top line and results with the adjusted positivity criteria are shown on the bottom line. TF = tumor fraction.

A. Cell line samples

Target TF (%)	ctDNA+/Total Replicates	Reproducibility (95% CI)
0.030 (6x LoD)	32/32	100.00% (89.11 - 100.00%)
0.020 (4x LoD)	20/20	100.00% (83.16 - 100.00%)
0.015 (3x LoD)	92/92	100.00% (96.07 - 100.00%)
0.010 (2x LoD)	79/79[a]	100.00% (95.44 - 100.00%)
0.005 (1x LoD)	79/80	98.75% (93.23 - 99.97%)
0.000	2/60<br>0/60[b]	96.67% (88.47 - 99.59%)<br>100.00% (94.03 - 100.00%)

[a]One replicate failed quality control.
[b]Adjusted positivity criteria results

B. Clinical samples

Sample	ctDNA+/Total Replicates
Positive – 0.015% TF (3x LoD)	5/5
Positive – 0.0075% TF (1.5x LoD)	5/5
Negative	0/5

S1G. Method Comparison

Methods: To assess the diagnostic accuracy of the MRD test, a method comparison study was performed using a modified TSO500 assay as the comparator test to detect ctDNA. Use of such a comparator is recommended by BloodPAC (Godsey, Silvestro et al. 2020). The TSO500 assay is designed as a liquid biopsy assay to inform therapy selection. LoD for the assay is 0.5% VAF for each interrogated gene (Illumina 2024). However, when summed across multiple genes to detect ctDNA, and by focusing only on known tumor-specific variants, the LoD for ctDNA detection (with ≥95% sensitivity) for the TSO500 assay was found to be 0.05% TF (data not shown). Samples from 76 patients with colorectal cancer (Table S1D1.A) who had unknown ctDNA status were processed in parallel through the MRD assay and TSO500 workflows. In total, 73 samples from 53 different patients met minimum cfDNA input requirements, passed quality control and returned valid results for both assays. All patients had a treatment-naïve blood sample, and 18 patients also had 1 (n = 16) or 2 (n = 2) post-treatment blood draws (Table S1D1.B). The agreement between the two tests was then calculated. The comparison of the TSO500 test results to the MRD assay was limited by the lower sensitivity for ctDNA detection of the TSO500 assay, with an LoD of 0.05% TF compared to the LoD of 0.005% TF established for the MRD assay.

Results: All four testing result possibilities occurred (both tests ctDNA positive, both ctDNA negative, one ctDNA positive and the other ctDNA negative, and vice-versa), with 17 of 73 (23%) samples showing discordant results between the two assays with the original positivity criteria and 13 of 73 (18%) with the adjusted positivity criteria (Table S1G.1).

The majority of the discordant results (15/17 and 11/13) were cases of a positive, ctDNA detected MRD assay result and a negative, ctDNA not detected TSO500 result. These 15 samples all had TFs below the established TSO500 ctDNA LoD of 0.05%TF. Further experimentation showed that a different set of patient samples positive by both assays remain positive by the MRD assay but flip to ctDNA negative by TSO500 when diluted to TFs below 0.05% (5 patients, 4 replicates per patient diluted to 0.015-0.007%TF), suggesting some of these discordant results are not false positives for the MRD assay (data not shown).

There were also two discordant samples for which the TSO500 assay gave a positive result, and the MRD assay gave a negative result. These two samples were different time

points from the same patient, and in both samples, the TSO500 positive result was caused by the same variant. The negative result from the MRD assay was due to the variant being excluded for being a CHIP variant. Whether the patient was in fact ctDNA-positive or gave a positive ctDNA result by TSO500 because of CHIP is unknown. To be conservative regarding the MRD assay performance, we treat the two results as false negatives here.

Conclusions: Using CRC patient samples with unknown ctDNA concentration, a method comparison of the MRD assay to TS0500 ctDNA detection showed perfect agreement among samples that had sufficient ctDNA to be consistently detected by both tests for both the original and revised positivity criteria. All but two of the discordant results were MRD assay positive and TSO500 assay negative and occurred for TFs below the TSO500 LoD. A potential CHIP variant that is not distinguished by the TSO500 test from somatic variants caused the two discordant TSO500 positive, MRD assay negative results, highlighting the utility of CHIP variant exclusion for ctDNA detection assays. Concordance for ctDNA not detected/negative samples increased approximately 25% with the adjusted positivity criteria.

Table S1G.1: Accuracy was determined by comparing MRD assay results with TSO500 assay results. Top line results are using original positivity criteria, bottom line uses adjusted positivity criteria. TF = tumor fraction. PPA = positive percent agreement; NPA = negative percent agreement; OPA = overall percent agreement.

MRD assay result:<br>TSO500 result:	Positive<br>Positive	Positive<br>Negative	Negative<br>Positive	Negative<br>Negative	PPA[a]<br>(95% CI)	NPA[a]<br>(95% CI)	OPA[a]<br>(95% CI)
All samples	41	15	2	15	95.3%<br>(84.5-98.7%)	50.0%<br>(33.2-66.8%)	76.7%<br>(65.8-84.9%)
	41	11	2	19	95.3%<br>(84.5-98.7%)	63.3%<br>(45.5 -78.1%)	82.2%<br>(71.9-89.3%)
Samples with TF > 0.05% (above TSO LoD)	14	0	0	0	100.0%<br>(78.5-100.0%)	-	-
	14	0	0	0	100.0%<br>(78.5-100.0%)	-	-
Samples with TF < 0.05% (below TSO LoD)	27	15	2	15	93.1%<br>(78.0-98.1%)	50.0%<br>(33.2-66.8%)	71.2%<br>(58.6-81.2%)
	27	11	2	19	93.1%<br>(78.0-98.1%)	63.3%<br>(45.5-78.1%)	78.0%<br>(65.9%, 86.6%)

[a]Assumes TSO500 results are reference values.

S1H. Analytical Specificity

Methods: Analytical specificity, defined as the true negative fraction of replicated results of pre-screened ctDNA-negative samples, was examined in samples from 11 CRC patients. Eleven patients with colorectal cancer (five stage II and six stage III) who were negative in a single result from both the TSO500 and MRD assays were utilized. Each sample was replicated four times with 30ng of cfDNA input.

Results: The eleven CRC patients had 110-200 targets in their designed patient-specific probe panels. Of the combined 44 replicates, 43 passed quality control. One of 43 had a positive ctDNA finding, giving an analytical specificity of 97.7% (95% CI: 88.0-99.9%). Reanalysis with the adjusted positivity criteria showed zero of 43 with a positive ctDNA detected test result, giving an analytical specificity of 100.0% (95% CI: 91.8–100.0%).

Conclusions: Using patient-derived clinical blood samples, the specificity of the MRD assay was 97.7% with the original positivity criteria and 100% with the adjusted positivity criteria.

References

Baden, J., C.-H. J. Lin, A. Anfora, J. Beer, K. Bisselou, J. Bungo, A. Corner, T. Danek, J. Dickey, J. H. Godsey, D. Johann, G. Jones, G. Karlin-Neumann, J. Larson, L. L. Jerry Lee, K. Meier, D. Merriam, M. Palomares, C. Pena, J. Rathbun, K. Rhodes, J. E. C. Rohrbach, A. Ryan, B. Saritas-Yildirim, M. Sausen, A. Silvestro, D. Stetson and L. C. Leiman (2024). "Generic Protocols for the AV of Tumor-Informed Circulating Tumor DNA Assays for Molecular Residual Disease: A Joint Consensus Recommendation of the BLOODPAC's MRD AV Working Group." submitted.

Croitoru, V. M., I. M. Cazacu, I. Popescu, D. Paul, S. O. Dima, A. E. Croitoru and A. D. Tanase (2021). "Clonal Hematopoiesis and Liquid Biopsy in Gastrointestinal Cancers." Front Med (Lausanne) **8**: 772166.

Feusier, J. E., S. Arunachalam, T. Tashi, M. J. Baker, C. VanSant-Webb, A. Ferdig, B. E. Welm, J. L. Rodriguez-Flores, C. Ours, L. B. Jorde, J. T. Prchal and C. C. Mason (2021). "Large-Scale Identification of Clonal Hematopoiesis and Mutations Recurrent in Blood Cancers." Blood Cancer Discov **2**(3): 226-237.

Godsey, J. H., A. Silvestro, J. C. Barrett, K. Bramlett, D. Chudova, I. Deras, J. Dickey, J. Hicks, D. J. Johann, R. Leary, J. S. H. Lee, J. McMullen, L. McShane, K. Nakamura, A. O. Richardson, M. Ryder, J. Simmons, K. Tanzella, L. Yee and L. C. Leiman (2020). "Generic Protocols for the Analytical Validation of Next-Generation Sequencing-Based ctDNA Assays: A Joint Consensus Recommendation of the BloodPAC's Analytical Variables Working Group." Clin Chem **66**(9): 1156-1166.

Illumina, I. (2024). "TruSight Oncology 500 ctDNA." Retrieved April 1, 2024, from https://www.illumina.com/content/dam/illumina/gcs/assembled-assets/marketing-literature/trusight-oncology-500-ctdna-data-sheet-m-gl-00843/trusight-oncology-500-ctdna-data-sheet-m-gl-00843.pdf.

Pierson-Perry, J. F., J. E. Vaks, A. P. Durham, C. Fischer, C. Gutenbrunner, D. Hillyard, M. V. Kondratovich, P. Ladwig and R. A. Middleberg (2012). Evaluation of Detection Capability for Clinical Laboratory Measurement Procedures; Approved Guideline—Second Edition. Wayne, PA, Clinical and Lboratory Safety Institute.

White, T., S. Szelinger, J. LoBello, A. King, J. Aldrich, N. Garinger, M. Halbert, R. F. Richholt, S. D. Mastrian, C. Babb, A. A. Ozols, L. J. Goodman, G. D. Basu and T. Royce (2021). "Analytic validation and clinical utilization of the comprehensive genomic profiling test, GEM ExTra((R))." Oncotarget **12**(8): 726-739.

S2. Swimmer plots

Figure S2.1: Swimmer plots for the 124 patients included in any analysis. (A) The 27 patients who recurred, from longest to shortest recurrence free interval, and (B) the 97 patients who did not recur, from longest to shortest follow-up.

A.

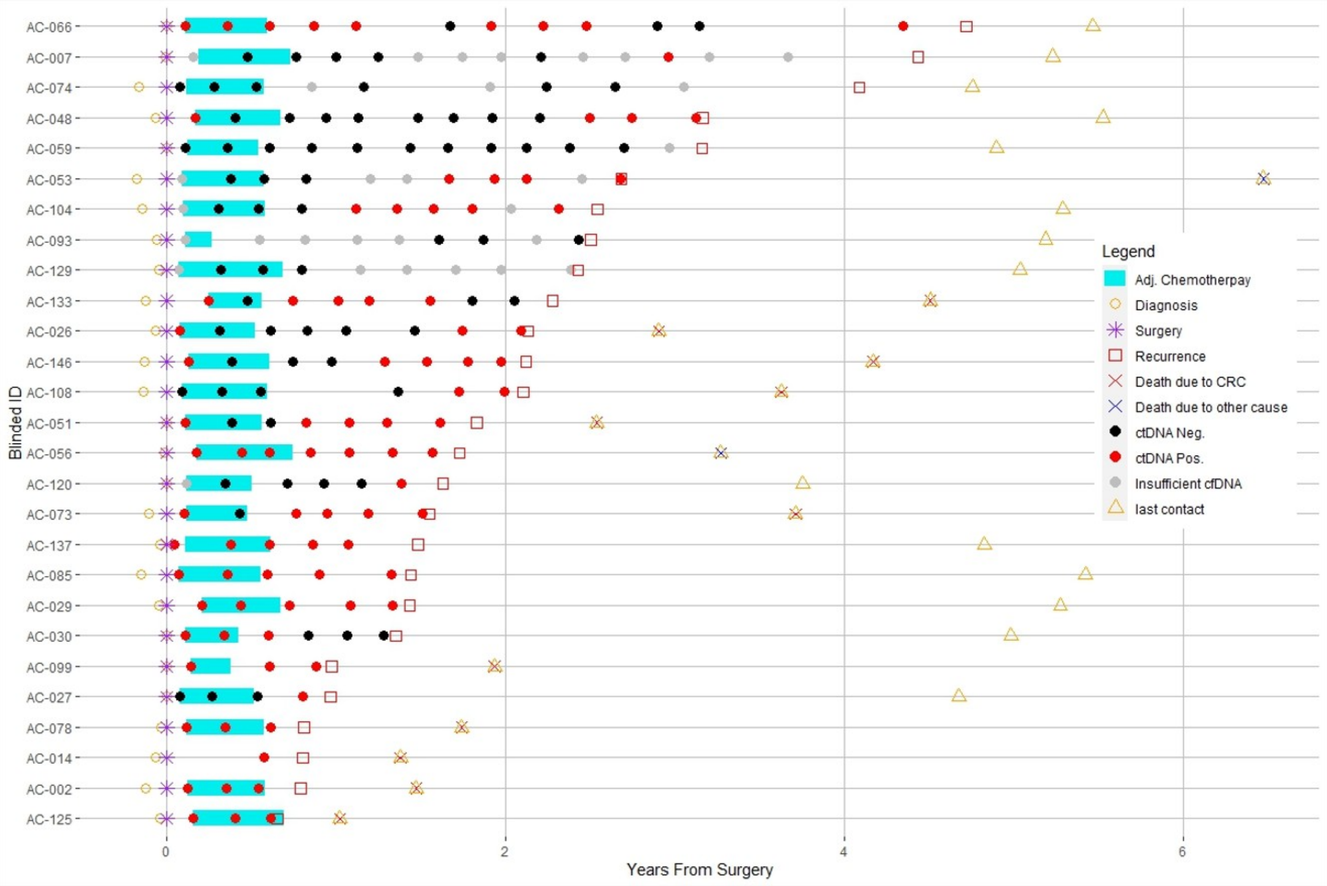

B.

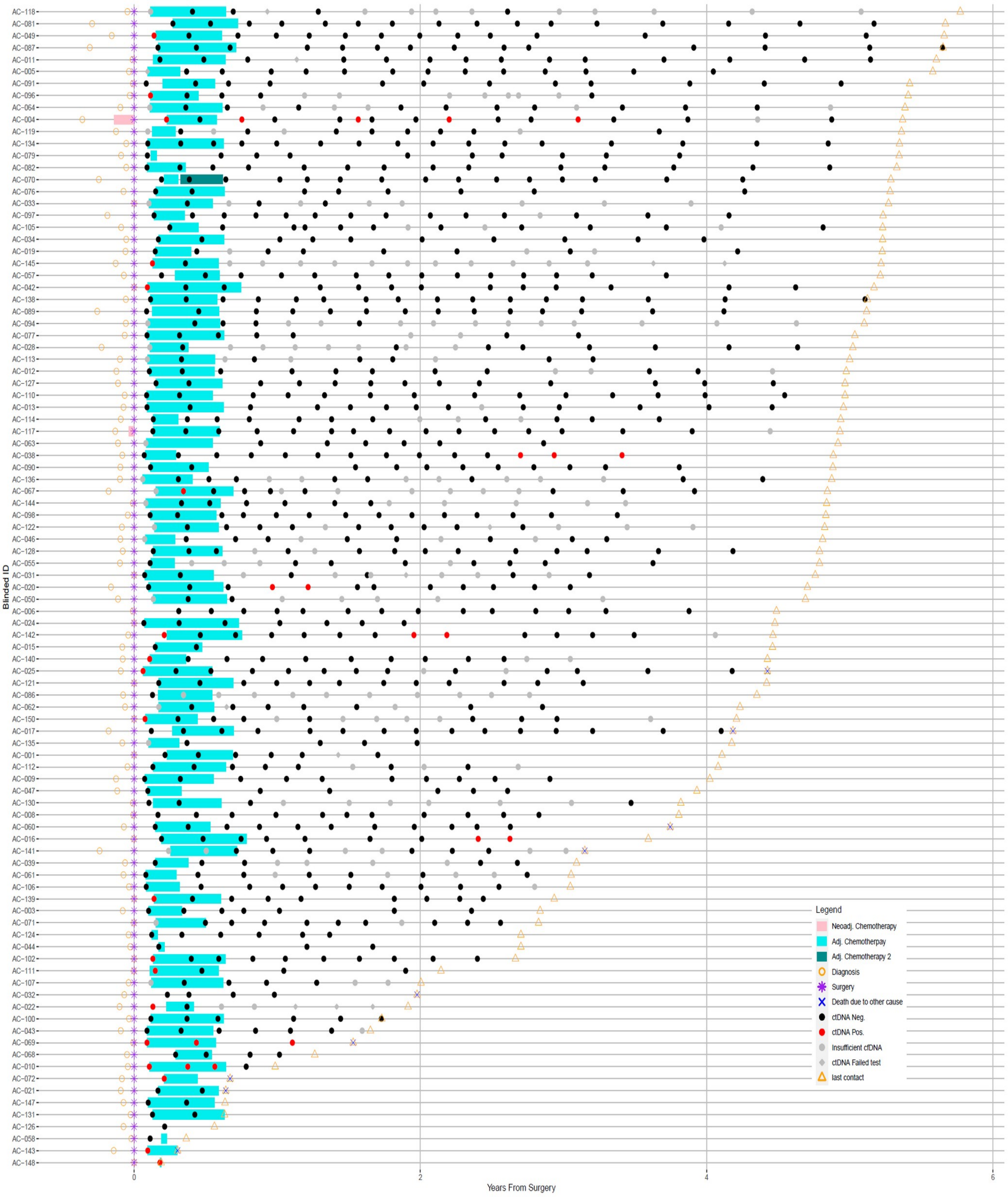

Blinded ID
Years From Surgery
0
2
4
6
Legend
Neoadj. Chemotherapy
Adj. Chemotherpay
Adj. Chemotherapy 2
Diagnosis
Surgery
Death due to other cause
ctDNA Neg.
ctDNA Pos.
Insufficient cfDNA
ctDNA Failed test
last contact

S3. Recurrence location and ctDNA positivity

Most sites of recurrence were represented by four or fewer events. However, liver and lung were more frequently represented. For patients with single sites of recurrence, liver had seven and lung had six recurrence events respectively. All seven liver recurrences and three of the lung recurrences were preceded by the most recent plasma sample result being ctDNA positive (Figure S3.1). The finding that patients with liver metastases tend to more often be ctDNA positive in the most recent sample compared to those with lung metastases has been observed in other studies. (Reinert, Henriksen et al. 2019; Reinert, Petersen et al. 2022; Henriksen, Demuth et al. 2024; Nakamura, Tsukada et al. 2024).

Figure S3.1: Number of recurrences that were immediately preceded by a ctDNA positive (ctDNA+) or negative (ctDNA-) result by location of metastatic event(s). “Other/Multiple” includes all metastatic events that were neither liver only nor lung only.

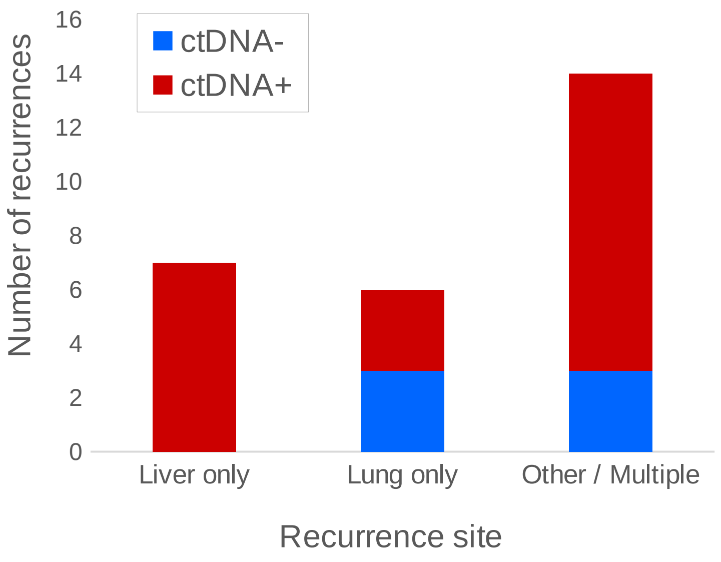

References

Henriksen, T. V., C. Demuth, A. Frydendahl, J. Nors, M. Nesic, M. H. Rasmussen, T. Reinert, O. H. Larsen, C. Jaensch, U. S. Love, P. V. Andersen, T. Kolbro, O. Thorlacius-Ussing, A. Monti, M. Gogenur, J. Kildsig, P. Bondeven, N. H. Schlesinger, L. H. Iversen, K. A. Gotschalck and C. L. Andersen (2024). "Unraveling the potential clinical utility of circulating tumor DNA detection in colorectal cancer-evaluation in a nationwide Danish cohort." Ann Oncol **35**(2): 229-239.

Nakamura, Y., Y. Tsukada, N. Matsuhashi, T. Murano, M. Shiozawa, Y. Takahashi, E. Oki, M. Goto, Y. Kagawa, A. Kanazawa, T. Ohta, A. Ouchi, H. Bando, H. Uchigata, C. Notake, H. Ikematsu and T. Yoshino (2024). "Colorectal Cancer Recurrence Prediction Using a Tissue-Free Epigenomic Minimal Residual Disease Assay." Clin Cancer Res.

Reinert, T., T. V. Henriksen, E. Christensen, S. Sharma, R. Salari, H. Sethi, M. Knudsen, I. Nordentoft, H. T. Wu, A. S. Tin, M. Heilskov Rasmussen, S. Vang, S. Shchegrova, A. Frydendahl

Boll Johansen, R. Srinivasan, Z. Assaf, M. Balcioglu, A. Olson, S. Dashner, D. Hafez, S. Navarro, S. Goel, M. Rabinowitz, P. Billings, S. Sigurjonsson, L. Dyrskjot, R. Swenerton, A. Aleshin, S. Laurberg, A. Husted Madsen, A. S. Kannerup, K. Stribolt, S. Palmelund Krag, L. H. Iversen, K. Gotschalck Sunesen, C. J. Lin, B. G. Zimmermann and C. Lindbjerg Andersen (2019). "Analysis of Plasma Cell-Free DNA by Ultradeep Sequencing in Patients With Stages I to III Colorectal Cancer." JAMA Oncol **5**(8): 1124-1131.

Reinert, T., L. M. S. Petersen, T. V. Henriksen, M. O. Larsen, M. H. Rasmussen, A. F. B. Johansen, N. Ogaard, M. Knudsen, I. Nordentoft, S. Vang, S. R. P. Krag, A. R. Knudsen, F. V. Mortensen and C. L. Andersen (2022). "Circulating tumor DNA for prognosis assessment and postoperative management after curative-intent resection of colorectal liver metastases." Int J Cancer **150**(9): 1537-1548.

S4. The association between ctDNA and CEA

Carcinoembryonic antigen (CEA) level is used in clinical practice to monitor recurrence and progression in stage II and III CRC. In this study, CEA level appeared to show a strong association with ctDNA at the sample level (Table S4.1). Abnormal CEA status (defined as normal, <2.5ng/ml for non-smokers or <5.0ng/ml for smokers, or abnormal, ≥2.5ng/ml for non-smokers or ≥5.0ng/ml for smokers) was 4.0 times more frequent in ctDNA positive samples compared to ctDNA negative samples. We did not perform a statistical test of this association as the samples were not independent (multiple samples were obtained from each patient). We also examined the association at the patient level by comparing CEA status (classified as: ≥1 abnormal versus always normal) and ctDNA status (classified as: ≥1 positive versus always negative) during surveillance. We observed a strong, statistically significant association between CEA and ctDNA, with abnormal CEA 3.3 times more frequent in patients with at least one positive ctDNA finding during surveillance (Fisher's Exact Test, $p < 0.001$; Table S4.2).

To gain further insight into the prognostic ability of CEA for recurrence in our study cohort, we calculated the lead time, specificity and sensitivity for recurrence during the surveillance period. We found that, for those patients who had an abnormal CEA result before recurrence, lead time (9.7 months) was similar to that observed for ctDNA, determined using the adjusted positivity criteria. However, only 14 patients who recurred had a preceding abnormal CEA result during surveillance, compared to 20 who had a ctDNA positive result during surveillance. CEA status thus showed lower sensitivity (60.9%) than ctDNA status (90.9%). Further, CEA status was abnormal for 15 patients who did not experience a recurrence, indicating a lower specificity (83.0%) than ctDNA status (94.3%).

Table S4.1: Sample level comparison of ctDNA and CEA status during surveillance period. ctDNA positivity appears to be strongly associated with abnormal CEA.

Number of samples (row %, col %)	CEA Normal	CEA Abnormal	Total samples
**ctDNA-**	783 (89.9%, 90.3%)	88 (10.1%, 60.3%)	871 (100.0%, 86.0%)
**ctDNA+**	84 (59.2%, 9.7%)	58 (40.8%, 39.7%)	142 (100.0%, 14.0%)
**Total samples**	867 (85.6%, 100.0%)	146 (14.4%, 100.0%)	1013 (100.0%, 100.0%)

Table S4.2: Patient level comparison of ctDNA and CEA status during surveillance period. ctDNA positivity is strongly associated with abnormal CEA (Fisher's Exact Test, $p < 0.001$).

Number of patients (row %, col %)	CEA Normal	CEA Abnormal	Total patients
**ctDNA-**	69 (82.1%, 86.3%)	15 (17.9%, 48.4%)	84 (100.0%, 75.7%)
**ctDNA+**	11 (40.7%, 13.8%)	16 (59.3%, 51.6%)	27 (100.0%, 24.3%)
**Total patients**	80 (72.1%, 100.0%)	31 (27.9%, 100.0%)	111 (100.0%, 100.0%)

S5. Results relaxing cfDNA input or sampling timeframe requirements

The MRD assay was validated with minimally 30ng of input cfDNA following library construction. Among the α-CORRECT samples, cfDNA was successfully extracted from 1297 (99%) samples, with 1029 (79%) yielding ≥30ng of DNA, 185 (14%) yielding ≥20 but <30ng of DNA, and 83 (6%) yielding <20ng of DNA. The 185 samples with 20-30ng of cfDNA were evaluated with the MRD assay using the adjusted positivity criteria and the analyses repeated to see how these lower cfDNA input samples might alter the conclusions. In addition, we investigated whether our conclusions might be altered if we set aside our sampling timeframe requirements, namely, PS sample collected 21-84 days after surgery, and PDT sample collected <180 days following definitive therapy. Relaxing these sampling requirements added 8 patients to the PS analyses (samples collected on days 17, 85, 90, 91, 99, 106, 114 and 210 after surgery) and 14 patients to the PDT analyses (samples collected on days 183, 196, 19g9, 202, 203, 205, 210, 214, 282, 309, 364, 376, 492, 766 after definitive therapy); there was no change in the number of patients in the surveillance analyses.

Adding the samples with cfDNA quantities ≥20ng and <30ng gave similar results to the analyses including only samples having cfDNA ≥30ng. Likewise, including patients outside of our prespecified time windows gave similar results to the analyses with these patients excluded (Tables S5.1A-C).

Table S5.1. Comparison of hazard ratios (HRs), sensitivity and specificity at the PS (A) and PDT (B) timepoints, and the surveillance period (C) when adding samples with cfDNA yields between 20 and 30ng, and when including patients with samples collected outside of prespecified time windows. Note that for the surveillance period, all patients with ≥1 sample after definitive therapy were already included in the analyses. PS = postsurgical, PDT = post-definitive therapy, CI = confidence interval.

A. PS timepoint.

Performance measure	Samples with ≥30ng cfDNA from patients within 21-84 day PS window	Samples with ≥20ng cfDNA from patients within 21-84 day PS window	Samples with ≥30ng cfDNA including patients outside 21-84 day PS window
HR (95% CI)	9.6 (3.2 – 29.5)	7.8 (3.2 – 29.5)	11.9 (4.0 - 35.6)
Sensitivity	77.8%	70.8%	81.0%
Specificity	80.3%	81.7%	81.7%

B. PDT timepoint.

Performance measure	Samples with ≥30ng cfDNA from patients within 180 day PDT window	Samples with ≥20ng cfDNA from patients within 180 day PDT window	Samples with ≥30ng cfDNA including patients outside 180 day PDT window
HR (95% CI)	16.7 (6.9 – 40.3)	15.4 (6.7 – 35.6)	15.7 (6.9 - 36.0)
Sensitivity	47.6%	41.7%	44.0%
Specificity	98.7%	98.8%	98.8%

C. Surveillance period.

Performance measure	Samples with ≥30ng cfDNA	Samples with ≥20ng cfDNA
HR (95% CI)	49.6 (16.6 – 148.3)	51.7 (17.3 – 154.5)
Sensitivity	90.9%	90.9%
Specificity	94.3%	93.5%
